# A Shift in Tissue Stiffness During Hippocampal Maturation Correlates to the Pattern of Neurogenesis and Composition of the Extracellular Matrix

**DOI:** 10.3389/fnagi.2021.709620

**Published:** 2021-07-30

**Authors:** Youngjae Ryu, Misato Iwashita, Wonyoung Lee, Kenji Uchimura, Yoichi Kosodo

**Affiliations:** ^1^Neural Regeneration Lab, Korea Brain Research Institute, Daegu, South Korea; ^2^Unit of Glycobiology Structure and Functions, CNRS—UMR 8576/University of Lille, Lille, France

**Keywords:** mechanical property, dentate gyrus, adult neurogenesis, atomic force microscopy, ultrasound microscopy, shear wave elasticity imaging, extracellular matrix, chondroitin sulfate proteoglycans

## Abstract

Aging changes the mechanical properties of brain tissue, such as stiffness. It has been proposed that the maintenance and differentiation of neural stem cells (NSCs) are regulated in accordance with extracellular stiffness. Neurogenesis is observed in restricted niches, including the dentate gyrus (DG) of the hippocampus, throughout mammalian lifetimes. However, profiles of tissue stiffness in the DG in comparison with the activity of NSCs from the neonatal to the matured brain have rarely been addressed so far. Here, we first applied ultrasound-based shear-wave elasticity imaging (SWEI) in living animals to assess shear modulus as *in vivo* brain stiffness. To complement the assay, atomic force microscopy (AFM) was utilized to determine the Young’s modulus in the hippocampus as region-specific stiffness in the brain slice. The results revealed that stiffness in the granule cell layer (GCL) and the hilus, including the subgranular zone (SGZ), increased during hippocampal maturation. We then quantified NSCs and immature neural cells in the DG with differentiation markers, and verified an overall decrease of NSCs and proliferative/immature neural cells along stages, showing that a specific profile is dependent on the subregion. Subsequently, we evaluated the amount of chondroitin sulfate proteoglycans (CSPGs), the major extracellular matrix (ECM) components in the premature brain by CS-56 immunoreactivity. We observed differential signal levels of CSPGs by hippocampal subregions, which became weaker during maturation. To address the contribution of the ECM in determining tissue stiffness, we manipulated the function of CSPGs by enzymatic digestion or supplementation with chondroitin sulfate, which resulted in an increase or decrease of stiffness in the DG, respectively. Our results illustrate that stiffness in the hippocampus shifts due to the composition of ECM, which may affect postnatal neurogenesis by altering the mechanical environment of the NSC niche.

## Introduction

Mechanical properties in the brain, including stiffness, are defined by various heterogeneous components, such as neurons, glial cells, vessels, the extracellular matrix (ECM), and interstitial fluids (Franze et al., 2013; Javier-Torrent et al., 2021). Several *in vitro* studies have shown that the proliferation, differentiation, and migration of neural stem cells (NSCs), progenitor cells, and post-mitotic neurons can be regulated depending on the stiffness of the surrounding environment (Saha et al., 2008; Leipzig and Shoichet, 2009; Rammensee et al., 2017). This has been considered as one of the fundamental cell fate-determining factors in neural development and neurological disorders (Barnes et al., 2017; Abuwarda and Pathak, 2020; Hall et al., 2021; Javier-Torrent et al., 2021).

NSCs are observed in specific areas in the mammalian brain, such as the subventricular zone (SVZ) and the dentate gyrus (DG) of the hippocampus, even in grown adults (Fuentealba et al., 2012; Bond et al., 2015). Among the NSCs observed in the adult brain, some are in an active state, while most are in a quiescent state (Urban et al., 2019). In the case of the DG, neurogenesis occurs from glia-like NSCs, which are located in the subgranular zone (SGZ). NSCs transform into neural progenitor cells, which subsequently differentiate into granule cells (Toni and Schinder, 2015; Toda et al., 2019). Previous studies have reported on heterogeneous tissue stiffness in the rodent hippocampus at the neonatal, juvenile, and adult stages (Elkin et al., 2010; Antonovaite et al., 2021). Based on such findings, it is deduced that the stiffness may be related to the niche of NSCs and their activity, including the maintenance and regulation of NSCs (Urban et al., 2019; Kobayashi and Kageyama, 2021). Indeed, it is reported that the mechanical stiffness of the stem cell niche in the SVZ can regulate NSC activity (Kjell et al., 2020). However, the following major questions remain to be addressed: (1) Does a shift in hippocampal stiffness correlate to the differentiation status of NSCs *in vivo*?; and (2) What is the principal component that determines tissue stiffness in the hippocampus?

As a technical aspect, atomic force microscopy (AFM) is generally employed to determine the Young’s modulus as the stiffness in local brain regions at both the embryonic (Iwashita et al., 2014; Nagasaka et al., 2016) and adult (Elkin et al., 2007; Luque et al., 2016; Antonovaite et al., 2018) stages. However, tissue slices are required to measure stiffness using AFM, which is a limitation for live animals. Recently, a method using ultrasound (US) has emerged to assess the shear modulus as stiffness in living animals. An acoustic radiation force (ARF) generated by a focused US beam induces shear waves inside the body, which allows for the measurement of the shear modulus *in vivo* (Doherty et al., 2013; Kuo et al., 2017). Using shear-wave elasticity imaging (SWEI), researchers showed that the shear modulus of a rodent brain is different depending on the brain region (Mace et al., 2011) and the age [4 vs. 11 months (M); Lay et al., 2019].

The hippocampus shows more abundant ECM expression levels than other regions (Dauth et al., 2016). It is reported that ECMs, such as hyaluronan and proteoglycan link protein 1 (Hapln1) and proteoglycans, are progressively upregulated over the aging process in rodents (Elkin et al., 2010; Vegh et al., 2014). Among those ECMs, particularly, chondroitin sulfate proteoglycans (CSPGs) are known to be abundant ECM components in developing nervous tissue and have been shown to be related to tissue stiffness in *Xenopus* brains (Koser et al., 2016). Moreover, CSPGs are highly expressed in neurogenic niches and promote hippocampal neurogenesis (Yamada et al., 2018). However, the role of CSPGs associated with hippocampal tissue stiffness has not been investigated so far.

Therefore, in this study, we examined the stiffness of the mouse brain at various time courses during maturation and observed the differentiation status of NSCs as well as immature neural cells. To suggest a correlation between the mechanical properties and pattern of neurogenesis, we observed subregions of the DG separately. Furthermore, we explored the transition and function of CSPGs in the hippocampus to find out their relevance for maturation-dependent changes in brain-tissue stiffness.

## Materials and Methods

### Experimental Animals

All the experiments were performed according to the guidelines of the Korea Brain Research Institute (KBRI) animal care and use committee. Animal experiments and related activities were approved by this committee (IACUC-20-00052). A total of 79 C57BL/6 mice were used in this experiment (purchased from OrientBio, South Korea, or bred at the KBRI). We used 38 male mice for AFM experiments, while both male and female mice were mixed randomly for other analyses. The day of birth was defined as postnatal day 0 (P0). We prepared the following time points for mice in this study: P1, P8, P15, P22, P31, 2–4 M, and 9–10 M. The animals were housed in a 12-h light/12-h dark cycle under controlled temperature (23–25°C). Food and water were supplied *ad libitum*.

### US-Based SWEI

Mice were anesthetized using sodium pentobarbital (50 mg/kg dose, intraperitoneal injection; Entobar, Hanlim Pharm., Seoul, South Korea). With the mouse in the prone position, the head of the animal was shaved appropriately, then the skull was gently opened from the Lambda to the Bregma suture point to expose the hippocampal region. After surgery, ultrasound gel (Aquasonic 100, Parker laboratories Inc., Fairfield, NJ, USA) was sufficiently placed onto the opened skull. For US imaging, we used PROSPECT T1 high-frequency US micro-imaging system (S-Sharp Corporation, Taiwan). First, we checked the correct position and the hippocampus area with B-mode imaging. Then, we attached an external radiation force transducer with an independent pulse generator and changed to the ARF mode to obtain the shear modulus. Information detailing the calculation of US-based elasticity can be found in the study of Mace et al. (2011) and the manufacturer^1^. US-SWEI was repeated four times using the same conditions for each animal. The parameters for the US navigation using the transducer probe were a broadband of 40.0 MHz, a 20-Hz frame rate, 30-μm resolution, and a dynamic range at 50 dB. For the ARF mode, we set the parameters at a 20.0-MHz push frequency, a 40.0-MHz detect frequency, and a 4,000 push cycle with a 200 μm duration and 190 μm intervals. After the US images were taken, mice were sacrificed by cervical dislocation.

### Acute Brain Slice Preparation and AFM Measurement

Mice were deeply anesthetized with intraperitoneal injections of sodium pentobarbital. Immediately after the brain was dissected out, we performed vibratome sectioning. The mouse brains were cut into 300-μm-thick whole coronal sections in ice-cold slicing artificial cerebrospinal fluid (aCSF; sucrose 175 mM, glucose 11 mM, NaCl 20 mM, KCl 3.5 mM, NaH_2_PO_4_ 1.25 mM, NaHCO_3_ 26 mM, and MgCl_2_ 1.3 mM) using a vibratome (VT1200S, Leica Biosystems, Richmond, IL, USA). Acute slices were incubated in slicing aCSF for 45 min on ice, then transferred to measuring-aCSF buffer (glucose 11 mM, NaCl 120 mM, KCl 3.5 mM, NaH_2_PO_4_ 1.25 mM, NaHCO_3_ 26 mM, MgCl_2_ 1.3 mM, and CaCl_2_ 2 mM) on a 35-mm poly-D-lysine-coated dish prior to indentation. The measurement of brain tissue stiffness was performed as previously described (Iwashita et al., 2020). Briefly, we used an AFM (Bioscope Resolve, NanoScope 9.4, Bruker, Billerica, MA, USA) mounted on an inverted microscope (ECLIPSE Ti2, Nikon, Japan). A tipless silicon cantilever with a 20-μm borosilicate bead (Novascan, Ames, IA, USA) was attached. The spring constant of the cantilever was calibrated using the thermal noise method in air. We chose cantilevers with the same spring constant (nominal value: 0.03 N/m; actual value: 0.07 N/m). The applied force was 10 nN. The specific parameters for the AFM measurement were a ramp size of 10 μm, a ramp rate of 1.0 Hz, a forward and reverse velocity of 20.0 μm/s, a sample Poisson’s ratio of 0.5, and a bead radius of 10.0 μm. The measurement was made under physiological conditions (37°C) for the acute slices. The force curves were acquired using the contact mode. To determine the right positions and region of interests (ROIs), bright field images were acquired by a CMOS camera (ORCA-Flash4.0, C13440-20CU, Hamamatsu, Japan).

### Manipulation of CSPGs

To induce the degradation of CSPGs, chondroitinase ABC (chABC; Sigma C3667) was used. Acute brain slices obtained from P8 were incubated with 2.5 unit/ml of chABC in measuring-aCSF buffer for 3 h at 25°C (Miller et al., 1997), then washed out with measuring-aCSF buffer to remove enzyme. Chondroitin sulfate (CS; Sigma C4384) was applied to acute brain slices in measuring-aCSF buffer (5 and 15 mg/ml) for 3 h at 25°C (Walz et al., 2002), then washed out with measuring-aCSF buffer to remove the CS that remained in the buffer. For the AFM measurement, the chABC or CS-treated slice was placed on 35-mm poly-D-lysine coated dish and kept in measuring-aCSF buffer prior to indentation. Brain slices untreated with either chABC or CS were used as a control for each experiment.

### Immunohistochemistry and Tissue Imaging

The P22, P31, and adult mice were deeply anesthetized with sodium pentobarbital and transcardially fixed with a 4% paraformaldehyde (PFA) solution. In the P1, P8, and P15 mice, the brain tissues were directly collected and immediately immersed in the PFA solution. The brain tissues were post-fixed in a 4% PFA solution for an additional 24 h at 4°C and soaked in a 20%-sucrose solution overnight. The tissues were cryosectioned into 30-μm-thick slices (Leica CM1520 cryostat, Leica Biosystems, Richmond, IL, USA). The tissue sections were incubated in a 2% BSA/0.1% Triton-X 100 solution for the blocking procedure, followed by incubation with diluted primary antibodies overnight. We used the following primary antibodies for immunostaining: anti-glial fibrillary acidic protein antibody (GFAP; mouse, 1:500, G3893, Merck Millipore, USA), anti-Sox2 antibody (rabbit, 1:300, AB5603, Merck Millipore, USA), anti-doublecortin antibody (DCX; rabbit, 1:500, #4604, Cell Signaling Technology, MA, USA), anti-Ki-67 antibody (Ki67; mouse, 1:500, #550609, BD Bioscience, USA), and anti-CSPG (CS-56; mouse, 1:300, ab11570). On the following day, the sections were incubated with secondary antibodies (Alexa Fluor 488 and 568 for each species-matched type, 1:500, Thermo Fisher Scientific, USA) and 4′,6-diamidino-2-phenylindole (DAPI; 1:1,000, Merck Millipore, USA) for 2 h. All tissue images were acquired using a confocal laser scanning microscope (TCS-SP8, Leica Biosystems, Richmond, IL, USA) and an upright confocal laser microscope (A1R-MP, Nikon, Japan). *Z*-stacked images were used for further analysis using five *z*-planes in 3–4 μm intervals.

### Data and Image Analysis

For US image analysis, we manually drew an ROI, and the shear modulus was calculated using PROSPECT software (S-Sharp Corporation, Taiwan). The targeted ROI areas were one of left or right hippocampus and the paired side of cortical region.

The AFM data were calculated using Nanoscope Analysis 1.9 software (Bruker, Billerica, MA, USA). The obtained force curves were adjusted to the baseline of each sample and analyzed to calculate Young’s modulus fit with the Hertzian model.

The brain tissue image analysis for cell counting and immunoreactivity of CS-56 was done using ImageJ (NIH, MD, USA) and Leica application suite X software (LAS X; Leica Biosystems, Richmond, IL, USA). The ROI area of the stained tissue was the DG in the hippocampus, and each sample of ROIs was analyzed using consequential tissue sections (mouse Bregma −1.70 mm to −2.20 mm).

### Statistical Analysis

All statistical analyses were performed using GraphPad Prism software (Version 9, GraphPad Software, San Diego, CA, USA). Statistically significant differences were determined by the student’s two-tailed *t*-test and a one-way or two-way analysis of variance (ANOVA) with *post hoc* Bonferroni test for correction of multiple comparisons. Results with *p* < 0.05 were considered to be statistically significant. The error bars in the figures denotes the standard error of the mean (SEM).

## Results

### Mechanical Properties of the Mouse Hippocampus and Cortex During Maturation as Evaluated by SWEI and AFM

**Figure 1:**
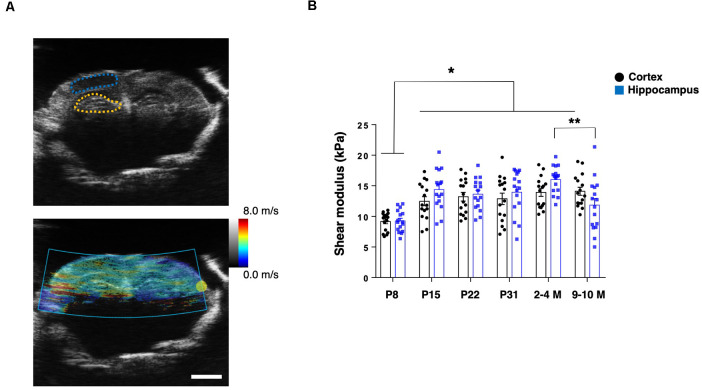
US-based shear-wave elasticity imaging (SWEI) to living post-natal mouse brain during maturation. **(A)** Top: representative B-mode image and regions of interest (ROIs; adult stage; blue-dotted line: somatosensory cortex; yellow-dotted line: hippocampus), bottom: shear wave propagation visualized by acoustic radiation force (ARF) mode. Scale bar: 2 mm. **(B)** Comparison of shear modulus obtained at various postnatal stages (black-round plots: cortex; blue-square plots: hippocampus). *p < 0.05, **p < 0.01.

To assess the mechanical properties of the brain tissue in live animals, we first applied US-based SWEI to mice of various ages, from P8 to grown adults (2–4 M and 9–10 M). The shear wave propagation generated by the ARF using a focused US beam shows reliable and consistent results to quantify the shear modulus as *in vivo* stiffness of the brain tissue, including humans and rodents (Mace et al., 2011; Tzschatzsch et al., 2018). From B-mode imaging, we identified the hippocampal and cortical (somatosensory) regions. Then, the ROIs were manually determined to calculate the shear modulus (Figure 1A). The shear modulus of the hippocampi and cortices in the adult mice (2–4 M) were 16.0 ± 0.6 kPa and 13.9 ± 0.7 kPa, respectively, which were in the comparable range of previously reported rodent brains (Mace et al., 2011; Lay et al., 2019). Notably, we found that the shear modulus of the P8 hippocampus (9.3 ± 0.5 kPa) and the cortex (9.2 ± 0.4 kPa) was significantly lower than at other ages (*n* = 4 for each group, two-way ANOVA; Figure 1B). Compared to 2–4 M mice, we observed a decreased shear modulus of the hippocampus in older mice (9–10 M; 11.8 ± 1.1 kPa) while a similar level in the cortex (14.1 ± 0.7 kPa), which was consistent with previous reporting (Lay et al., 2019). However, we did not observe significant differences between the shear modulus of the hippocampal and cortical regions in any groups.

**Figure 2:**
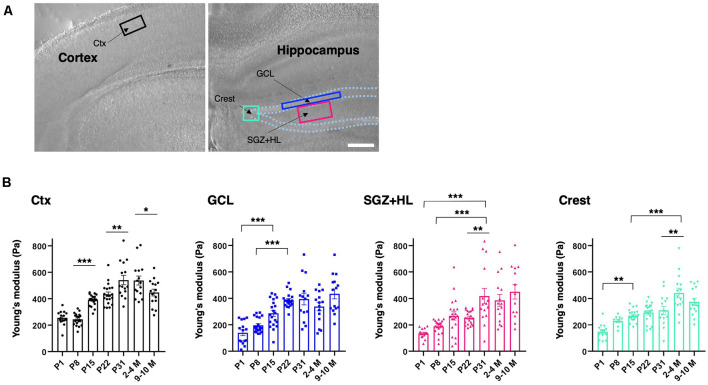
Spatiotemporal shift in stiffness in the dentate gyrus (DG) as examined by atomic force microscopy (AFM).** (A)** The representative cortical and hippocampal areas under a bright field microscope (P8 stage). Rectangles indicate the ROIs for AFM measurement. Ctx, cortex; GCL, granule cell layer; SGZ, subgranular zone; HL, hilus. Scale bar: 200 μm. **(B)** The bar graph with plots presenting Young’s modulus in various subregions (black-round plots: cortex; blue-square plots: GCL; magenta-triangle plots: SGZ+HL; green-inverted triangle plots: crest of the DG). *p < 0.05, **p < 0.01, ***p < 0.001.

To determine the subregional stiffness of the hippocampus, an AFM measurement was performed along the maturation course. AFM analysis is widely used for measuring the Young’s modulus for stiffness in biological samples, including brain tissue, and is considered a current gold standard method (see “Introduction” section). Since AFM-based stiffness measurement is hardly applicable to live animals, we prepared *ex vivo* acute brain slices to acquire the Young’s modulus from the neonatal (P1) to adult stages (2–4 M and 9–10 M). For indentation, we determined ROIs in the DG of the hippocampus where newborn neurons are observed in adult mice (Figure 2A), and then subdivided the DG regions into the granule cell layer (GCL), the hilus including the SGZ (SGZ+HL), and the crest (Amaral, 1978; MacLennan et al., 1998). The Young’s modulus of the somatosensory region in the cortex was measured as a reference. We found that the stiffness in all subregions of the hippocampi and cortices tested here increased during maturation (*n* = 3 for P1, P31, 2–4 M, and 9–10 M, *n* = 4 for P8, P15, and P22, one-way ANOVA; Figure 2B and Table 1). A slight decrease in Young’s modulus was observed in the cortex in older mice (9–10 M). Notably, we recognized that the stages when stiffness reached a plateau differed among subregions in the hippocampus. In the case of the GCL, the stiffness increased gradually from P1 (140 ± 22 Pa) to P22 (386 ± 12 Pa) with significant differences. There were no significant changes from P22 to 9–10 M. Similarly, in the SGZ+HL and crest, stiffness started around 140 Pa at P1, significantly increased along with age, and reached its peak at P31 (418 ± 58 Pa) for SGZ+HL or 2–4 M (442 ± 37 Pa) for the crest (Figure 2B). Taking the result of the US-based SWEI and AFM analysis together, it can be commonly indicated that the stiffness of hippocampal tissue increases from the neonatal to the early adult stage. Furthermore, time courses of the shift in mechanical environments have specific profiles among subregions in the DG along the maturation course.

**Table 1:** Summary of the atomic force microscopy (AFM) measurements.

	P1 (*n* = 3)	P8 (*n* = 4)	P15 (*n* = 4)	P22 (*n* = 4)	P31 (*n* = 3)	2–4 M (*n* = 3)	9–10 M (*n* = 3)
Cortex	255 ± 14 Pa (*15*)	244 ± 10 Pa (*20*)	390 ± 9 Pa (*20*)	433 ± 18 Pa (*20*)	541 ± 35 Pa (*15*)	537 ± 34 Pa (*15*)	447 ± 26 Pa (*15*)
GCL	140 ± 22 Pa (*15*)	196 ± 12 Pa (*20*)	287 ± 23 Pa (*20*)	386 ± 12 Pa (*20*)	394 ± 44 Pa (*14*)	337 ± 31 Pa (*15*)	434 ± 37 Pa (*15*)
SGZ+HL	136 ± 9 Pa (*15*)	188 ± 11 Pa (*20*)	268 ± 31 Pa (*20*)	255 ± 12 Pa (*20*)	418 ± 58 Pa (*15*)	386 ± 45 Pa (*15*)	451 ± 55 Pa (*13*)
Crest	149 ± 15 Pa (*14*)	230 ± 13 Pa (*9*)	269 ± 10 Pa (*20*)	293 ± 16 Pa (*20*)	310 ± 30 Pa (*15*)	442 ± 37 Pa (*15*)	374 ± 27 Pa (*15*)

*The “*n*” indicates the number of animals as well as the number of measured slices (one slice from each animal). **Italicized figures: number of measured points

### Cell Type Populations in the Specific Neural Differentiation Phase at Various Postnatal Stages in the DG of the Hippocampus

**Figure 3:**
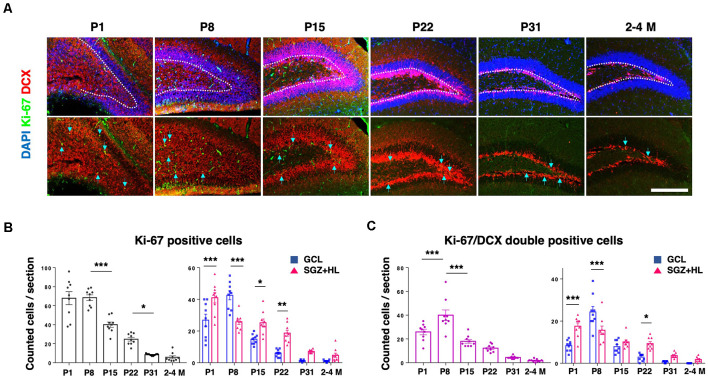
The expression of Ki-67 and DCX in the DG. **(A)** Representative immunostaining images for Ki-67 (green), DCX (red), and DAPI (blue) at various postnatal stages. Arrows indicate Ki-67/DCX-double-positive cells. Scale bar: 200 μm. **(B)** Left: the number of Ki-67-positive cells in the DG in each section. Right: comparison of Ki-67-positive cells in the GCL and SGZ+HL area. **(C)** Left: the number of Ki-67/DCX-double-positive cells in the DG in each section. Right: comparison of the double-positive cells in the GCL and SGZ+HL area (blue-square plots: GCL; magenta-triangle plots: SGZ+HL). *p < 0.05, **p < 0.01, ***p < 0.001.

Next, we analyzed the activity of NSCs using neurogenesis markers to address the relationship with mechanical characteristics of the hippocampus. Using PFA-fixed brain sections, we examined the expression of Ki-67, a proliferative cell marker, and doublecortin (DCX) for defining immature neuronal cells (Figure 3A; Rao and Shetty, 2004). The number of Ki-67-positive cells in the DG was higher at neonatal stages (P1 and P8), then decreased during maturation. The Ki-67-labeled cells were mostly downregulated by the P31 stage, showing a similar level to adult brains (*n* = 3 for each group, left graph: one-way ANOVA, right graph: two-way ANOVA; Figure 3B). The number of Ki-67/DCX-double-positive cells peaked at P8, then decreased in later stages (left graph: one-way ANOVA, right graph: two-way ANOVA; Figure 3C). It is noteworthy that the Ki-67-positive cells and Ki-67/DCX-double-positive cells were located significantly more in the SGZ+HL than in the GCL at P1, but the population densities reversed at P8. After the P8 stage, the Ki-67-positive and Ki-67/DCX-double-positive cells were located more in the SGZ+HL than the GCL, or were similar between the two regions.

**Figure 4:**
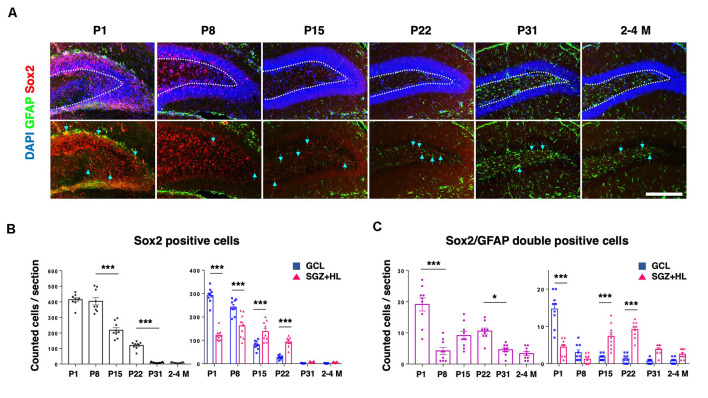
The Sox2- and GFAP-positive cells in the DG. **(A)** Representative immunostaining images for Sox2 (red), GFAP (green), and DAPI (blue) at various postnatal stages. Arrows indicate Sox2/GFAP-double-positive cells. Scale bar: 200 μm. **(B)** Left: the number of Sox2-positive cells in the DG in each section. Right: Comparison of Sox2-positive cells in the GCL and SGZ+HL area. **(C)** Left: the number of Sox2/GFAP-double-positive cells in the DG in each section. Right: comparison of the double-positive cells in the GCL and SGZ+HL area (blue-square plots: GCL; magenta-triangle plots: SGZ+HL). *p < 0.05, ***p < 0.001.

In order to assess the population of NSCs in the DG, we counted GFAP and Sox2 in each postnatal group (Figure 4A). Sox2 is a transcription factor expressed in multipotent NSCs (Ellis et al., 2004), while primitive radial glia-like NSCs are double positive for GFAP and Sox2 (Sachewsky et al., 2014). As expected, the total population of Sox2-labeled cells in the DG gradually decreased after P1. At the P1 and P8 stages, the number of Sox2-positive cells in the GCL was significantly higher than the SGZ+HL. Notably, it was observed that the number of Sox2-positive cells in the GCL became fewer than in the SGZ+HL at P15 and P22 (*n* = 3 for each group, left graph: one-way ANOVA, right graph: two-way ANOVA; Figure 4B). We found that the number of Sox2/GFAP-double-positive cells in the DG was lowest at P8 among the early postnatal stages (P1 to P22). Similar to the pattern of Ki67/DCX-double-positive cell populations, the Sox2/GFAP-double-positive cells were significantly increased in the SGZ+HL compared to the GCL after P8 (*n* = 3 for each group, left graph: one-way ANOVA, right graph: two-way ANOVA; Figure 4C).

Regarding the adult stages tested in this study (2–4 M and 9–10 M), neither the staining patterns nor the quantification of used markers (Ki-67/DCX or Sox2/GFAP) were different between the groups (only data for 2–4 M is shown). Our quantitative analysis indicates that postnatal neurogenesis in the DG occurs differently among the hippocampal subregions during brain maturation, especially in the stages between P1 and P22.

### The Role of CSPGs in Determining Tissue Stiffness in the DG During Hippocampal Maturation

**Figure 5:**
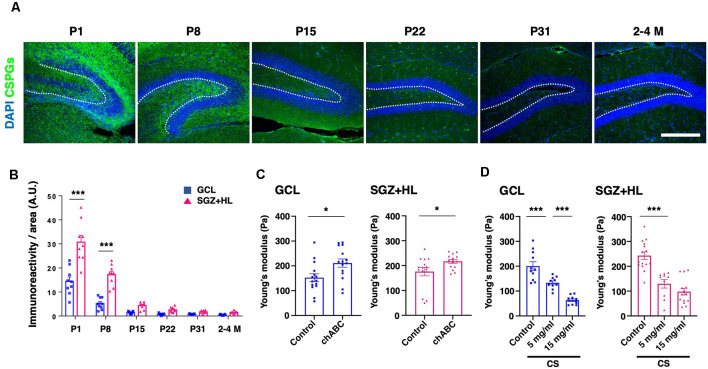
The transition of CSPGs in the DG during maturation and the shift in stiffness by CSPG manipulation. **(A)** Representative immunostaining images for CSPGs using CS-56 antibody (green) and DAPI (blue) at various postnatal stages. The white-dotted lines represent the border between the GCL and SGZ+HL area. Scale bar: 200 μm. **(B)** The bar graph with plots presents a quantitative analysis of the immunoreactivity of the CSPGs in the GCL and SGZ+HL area (blue-square plots: GCL; magenta-triangle plots: SGZ+HL). **(C)** The bar graph with plots presents a quantitative analysis of the stiffness in the GCL and SGZ+HL area treated with chABC (blue-square plots: GCL; magenta-triangle plots: SGZ+HL). **(D)** The bar graph with plots presents a quantitative analysis of the stiffness in the GCL and SGZ+HL area treated with CS. Final concentrations supplied in the buffer are indicated. *p < 0.05, ***p < 0.001.

To explore the factors accounting for the shift in brain tissue stiffness during maturation, we examined the immunoreactivity of CS-56 antibody, which recognizes the specific oligosaccharide structure containing both C6-sulfation and C4-sulfation in CSPGs (Miyata and Kitagawa, 2016; Figure 5A). We found that CS-56 immunoreactivity was significantly higher in the SGZ+HL than the GCL at the P1 and P8 stages (*n* = 3 for each group, two-way ANOVA; Figure 5B), exhibiting a gradual decrease during maturation toward the adult stages (only data for 2–4 M is shown). The result implies that CS-56-positive CSPGs are enriched in the early postnatal stages when the hippocampal tissue shows softer stiffness and has more NSC population in the DG.

For further investigation of the link between CSPGs and stiffness in the DG, we manipulated the molecular functions of CSPGs in tissue combined with the stiffness measurement by AFM. First, we applied chABC to enzymatically degrade CSPGs in the slice of P8 brain at the stage when a higher level of CS-56 immunoreactivity was observed. Subsequent AFM measurement revealed that stiffness in both the GCL and SGZ+HL was significantly increased in chABC-treated brain slices (*n* = 3 for each group, unpaired *t*-test; Figure 5C and Table 2A). Next, we treated the P8 brain slices with excessive CS, as performed in the developing *Xenopus* brain to decrease tissue stiffness (Koser et al., 2016). We observed that stiffness in both the GCL and SGZ+HL was decreased in CS-treated brain slices in a concentration-dependent manner (*n* = 3 for control and 15 mg/ml of CS and *n* = 2 for 5 mg/ml of CS, one-way ANOVA; Figure 5D and Table 2B). These results imply that CSPGs serve a critical role in determining tissue stiffness in the DG during hippocampal maturation.

**Table 2:** Summary of the AFM measurements with chondroitin sulfate proteoglycan (CSPG) manipulation.

(A) Young’s modulus of the chABC-treated tissue					
GCL		SGZ+HL			
Control (*n* = 3)	chABC (*n* = 3)	Control (*n* = 3)	chABC (*n* = 3)
152 ± 16 Pa (*15*)	211 ± 17 Pa (*15*)	176 ± 17 Pa (*15*)	217 ± 7 Pa (*15*)
**(B) Young’s modulus of CS-treated tissue**					
**GCL**			**SGZ+HL**		
Control (*n* = 3)	5 mg/ml (*n* = 2)	15 mg/ml (*n* = 3)	Control (*n* = 3)	5 mg/ml (*n* = 2)	15 mg/ml (*n* = 3)
200 ± 17 Pa (*11*)	133 ± 8 Pa (*10*)	63 ± 5 Pa (*11*)	243 ± 15 Pa (*15*)	129 ± 17 Pa (*10*)	98 ± 14 Pa (*14*)

*The “*n*” indicates the number of animals as well as the number of measured slices (one slice from each animal). **Italicized figures: number of measured points

## Discussion

In the present study, we focused on the mechanical properties of the hippocampus, especially stiffness in the DG along with the time course of tissue maturation at various postnatal stages. For this purpose, we used US-based SWEI and AFM to quantify the *in vivo* shear modulus and the *ex vivo* subregional Young’s modulus, respectively. Also, we compared the population and differentiation of NSCs in subregions of the hippocampus at the time points when stiffness measurements were taken. Furthermore, we addressed the transition profile and role of CSPGs as the major factor in determining hippocampal tissue stiffness. Together, our work uncovered the relevance between spatiotemporal stiffness and the significant potency of the ECM, which may regulate NSC activity in the hippocampus.

SWEI allows for assessment of the shear modulus as intact tissue stiffness using ARF generated by a push transducer. Recently, SWEI has been widely used for evaluating stiffness *in vivo*, including musculotendinous tissues (Kammoun et al., 2019). Our results pertaining to cortical and hippocampal tissue stiffness are in a comparable range to other studies that used SWEI in adult rodents (within 10 ± 7 kPa; Mace et al., 2011; Lay et al., 2019). However, these results are not of the same order of magnitude as the Young’s modulus acquired by AFM-based indentation assessment for the same tissue (ranged within 1 ± 0.5 kPa), which are in agreement with the values of previous reports on post-natal mouse brains (Elkin et al., 2010; Iwashita et al., 2020; Antonovaite et al., 2021). A possible way to account for this difference is that amputation of stiffer tissue components, such as vessels and axons, by slice preparation for AFM measurement may result in decreased stiffness compared to the intact brain. Besides, because of its softness and high deformability for brain tissue, the application of a nonlinear model combined with an inverse analysis might be appropriate for calculating the shear modulus in SWEI measurements (Jiang et al., 2015). In this study, initially, we used SWEI to detect brain tissue stiffness in living animals, although we could not specify subregions of the hippocampus due to insufficient resolution. We then utilized an AFM measurement by preparing *ex vivo* brain slices to evaluate specific regional stiffness. Of note, the SWEI assay indicates that the overall shear modulus in the hippocampus region was significantly decreased in the older brain (9–10 M) compared to the younger brain, while the tendency of the Young’s moduli determined by AFM varied dependent on the subregions of the DG (Figures 1, 2). Despite these points, both approaches commonly showed the overall increase of tissue stiffness during hippocampal maturation, which is consistent with previous studies on rodent brains (Elkin et al., 2010; Antonovaite et al., 2021). It is reported that the GCL of adult mice shows lower stiffness relative to the other parts of the hippocampus (Antonovaite et al., 2018). Our data are largely in agreement with this report on the adult brain, although we could not detect statistically significant differences. Notably, we further identified that the stiffness of the GCL was higher (386 ± 12 Pa) than that of the SGZ+HL area (255 ± 12 Pa) at P22 (Figure 2 and Table 1). This result suggests that there is a dynamic conversion in stiffness in particular subregions during brain maturation. We assume that it can be due to: (1) vigorous alteration of neural cell types and populations by cellular differentiation as well as migration with morphological changes (Abuwarda and Pathak, 2020); or (2), production or degradation of specific ECM components (Walma and Yamada, 2020, and this study).

Neurogenesis in the DG of the hippocampus is observed throughout the lifetime of mammals, and the differentiation of NSCs in the hippocampus can be dependent on the tissue environments, including mechanical cues (Urban et al., 2019; Kobayashi and Kageyama, 2021). Indeed, several *in vitro* studies have shown that mechanical stiffness affects the migration and differentiation of NSCs (see “Introduction” section). Here, our data show that the neurogenesis in the DG decreases during maturation; meanwhile, the hippocampal tissue stiffness increases. Particularly, we observed that the populations of NSCs (Sox2/GFAP-double-positive cells; Figure 4) and proliferative immature neural cells (Ki-67/DCX-double-positive cells; Figure 3) in the SGZ+HL compared to the GCL area show dynamically differential patterns from the neonatal to the adult stage. As described above, the SGZ+HL showed lower stiffness than that of the GCL area especially at P22 (Figure 2 and Table 1). Considering that NSCs are more differentiated into glial cells on a harder substrate than a soft substrate (Saha et al., 2008; Tse and Engler, 2011), the significantly greater populations of Sox2/GFAP- and Ki-67/DCX-double-positive neural cells in the SGZ+HL compared to the GCL area at P22 but not P31 and adult stages (Figures 3, 4) may be well explained by the preference of softer regions for neural cells. Taking the previous results and our study together, we raise a possibility that the differences in stiffness among the hippocampal subregions could be one of the factors determining the pattern of transition/migration of the NSCs and the immature neural-cell population.

Our approach to manipulating the function of CSPGs in brain slices revealed the importance of ECM composition to determining tissue stiffness. The enzymatic digestion of CSPGs induced the stiffening of tissue, while the addition of CS had the opposite effect. These results fit well into the temporal profiling of the ECM and the shear modulus as well as Young’s modulus; that is, a higher level of CS-56-positive CSPGs correlated to lower brain stiffness at early postnatal stages in the hippocampus. Among the subregions of the DG, the CSPG level in the SGZ+HL was higher than that of the GCL in all groups, although the stiffness between the SGZ+HL and GCL was essentially similar during hippocampal maturation except at P22. We consider the possibility that the other ECMs of perineuronal nets, such as hyaluronan and Hapln1, may be involved in determining stiffness in the hippocampus (Vegh et al., 2014; Richter et al., 2018). Alternatively, not only the amount of CSPGs but also the difference in the mode of chondroitin sulfation may affect the mechanical microenvironment in tissue. Indeed, it has been shown that chondroitin C6-sulfation is dominant early in the postnatal period, while the degree of C4-sulfation increases during the maturation of the mouse brain (Miyata et al., 2012). Further systematic analyses to clarify the molecular constituent of ECMs and their origins in tissue, as well as cell-types, are required.

By coupling the measurement of mechanical properties in hippocampal tissue and quantitative cellular characterization at the various postnatal stages, our data suggest a correlation between the tissue’s micro-mechanical stiffness and the composition of the ECMs, which may have regulatory effects on the activity of NSCs. It is worth noting that the changes of NSCs/neural immature cell populations and stiffness in the subregions of the DG were observed during the early postnatal period. Whether the same or different principle exists in the case of a much older brain or neurodegenerative disease is still an open question. We expect that further related studies can reveal regulatory mechanisms of *in vivo* neurogenesis associated with the modulation of brain tissue stiffness.

## Data Availability Statement

The original contributions presented in the study are included in the article, further inquiries can be directed to the corresponding author.

## Ethics Statement

The animal study was reviewed and approved by Korea Brain Research Institute Animal Care and Use Committee.

## Author Contributions

YR and YK: study design and conceptualization. YR: manuscript writing. YR, MI, and YK: manuscript editing. Experiments: YR for US-SWEI, AFM, and neural differentiation analyses, MI for AFM and CSPG manipulations, WL for US-SWEI. YR, MI, and KU: resources. YR, MI, and YK: data validation. YK: project administration. All authors contributed to the article and approved the submitted version.

## Conflict of Interest

The authors declare that the research was conducted in the absence of any commercial or financial relationships that could be construed as a potential conflict of interest.

## Publisher’s Note

All claims expressed in this article are solely those of the authors and do not necessarily represent those of their affiliated organizations, or those of the publisher, the editors and the reviewers. Any product that may be evaluated in this article, or claim that may be made by its manufacturer, is not guaranteed or endorsed by the publisher.

## Footnotes

^1^ https://www.s-sharp.com
